# Supplementary material for: Integrating CT Radiomics and Clinical Features to Optimize TACE Technique Decision-Making in Hepatocellular Carcinoma
Source: Cancers (Basel). 2025 Mar 5;17(5):893. doi: 10.3390/cancers17050893 (PMC11899091; doi:10.3390/cancers17050893)
Supplement: Supplementary file 1 [file cancers-17-00893-s001.zip › cancers-3478591-supplementary.pdf]

Table S1. Analyzed predictor variables for response analysis

Clinical and laboratory parameters

Sex
Age
WBC
Hb
Platelets
Quick
INR
pTT
Bilirubin
AST
ALT
GGT
AP
LDH
Albumin
AFP
Etiology of liver disease
ECOG
BCLC
Sum of target lesion diameter
Indication for TACE

Radiomic features

Energy
Entropy
InterquartileRange
Kurtosis
Maximum
MeanAbsoluteDeviation
Mean
Median
Minimum
Range
RobustMeanAbsoluteDeviation
RootMeanSquared
Skewness
TotalEnergy
Uniformity
Variance
Autocorrelation
ClusterProminence
ClusterShade
ClusterTendency
Contrast
Correlation
DifferenceAverage
DifferenceEntropy
DifferenceVariance
Id

Idm
Idmn
Idn
Imc1
Imc2
InverseVariance
JointAverage
JointEnergy
JointEntropy
MCC
MaximumProbability
SumAverage
SumEntropy
SumSquares
DependenceEntropy
DependenceNonUniformity
DependenceNonUniformityNormalized
DependenceVariance
GrayLevelNonUniformity
GrayLevelVariance
HighGrayLevelEmphasis
LargeDependenceEmphasis
LargeDependenceHighGrayLevelEmphasis
LargeDependenceLowGrayLevelEmphasis
LowGrayLevelEmphasis
SmallDependenceEmphasis
SmallDependenceHighGrayLevelEmphasis
SmallDependenceLowGrayLevelEmphasis
GrayLevelNonUniformity
GrayLevelNonUniformityNormalized
GrayLevelVariance
HighGrayLevelRunEmphasis
LongRunEmphasis
LongRunHighGrayLevelEmphasis
LongRunLowGrayLevelEmphasis
LowGrayLevelRunEmphasis
RunEntropy
RunLengthNonUniformity
RunLengthNonUniformityNormalized
RunPercentage
RunVariance
ShortRunEmphasis
ShortRunHighGrayLevelEmphasis
ShortRunLowGrayLevelEmphasis
GrayLevelNonUniformity
GrayLevelNonUniformityNormalized
GrayLevelVariance
HighGrayLevelZoneEmphasis
LargeAreaEmphasis
LargeAreaHighGrayLevelEmphasis
LargeAreaLowGrayLevelEmphasis
LowGrayLevelZoneEmphasis

SizeZoneNonUniformity
SizeZoneNonUniformityNormalized
SmallAreaEmphasis
SmallAreaHighGrayLevelEmphasis
SmallAreaLowGrayLevelEmphasis
ZoneEntropy
ZonePercentage
ZoneVariance
Busyness
Coarseness
Complexity
Contrast
Strength

Abbreviations: AFP, Alpha-fetoprotein; ALP, Alkaline phosphatase; ALT, Alanine transaminase; AST, Aspartate transaminase; BCLC, Barcelona Clinic Liver Cancer (stage); ECOG, Eastern Cooperative Oncology Group (performance status); GGT, Gamma-glutamyl transferase; Hb, Hemoglobin; INR, International normalized ratio; LDH, Lactate dehydrogenase; pTT, Partial thromboplastin time; TACE, Transarterial chemoembolization; WBC, White blood cells
